# A Novel Echocardiographic Index (Modified-Left-Atrium-to-Aorta Ratio) for Quantifying Left Atrial Size and Differentiating Stages in Dogs with Myxomatous Mitral Valve Disease

**DOI:** 10.3390/ani15121820

**Published:** 2025-06-19

**Authors:** Minsuk Kim, Minwoong Seo, Chul Park

**Affiliations:** Department of Veterinary Internal Medicine, College of Veterinary Medicine, Jeonbuk National University, Iksan 54596, Jeonbuk, Republic of Korea; justin1866@jbnu.ac.kr (M.K.); minung0204@gmail.com (M.S.)

**Keywords:** canine, echocardiography, left atrium, myxomatous mitral valve disease

## Abstract

Myxomatous mitral valve disease (MMVD) is the most common heart disease in small-breed dogs, and accurate assessment of the left atrial (LA) size is essential for diagnosis and management. The traditional echocardiographic method, LA-to-Aorta (LA/Ao) ratio, is widely used, but evaluates LA size in only one view. This study introduces a novel index—the modified-LA/Ao (M-LA/Ao) ratio—which combines two echocardiographic views to better reflect the true LA size. This study retrospectively analyzed thoracic radiographs and echocardiograms from 136 dogs, including healthy controls and dogs with MMVD classified into stages B1, B2, and C according to ACVIM guidelines. The ability of the M-LA/Ao ratio is compared to conventional indices using correlation analysis and receiver operating characteristic (ROC) curves. The M-LA/Ao ratio showed a strong correlation with existing indices and slightly improved discrimination between ACVIM stages B1 and B2, although performance between stages B2 and C was similar to the LA/Ao ratio. Intra- and interobserver variability were also acceptable. Our findings suggest that the M-LA/Ao ratio may provide a practical and sensitive method to evaluate LA enlargement in early-stage MMVD, helping clinicians detect subtle cardiac remodeling before progression to advanced disease.

## Simple Summary

Myxomatous mitral valve disease is the most common heart disease in small dogs. In this disease, the left atrium becomes enlarged as the disease gets worse. Veterinarians commonly use two-dimensional echocardiography to measure this enlargement, but the traditional method relies on a single angle and might miss some changes in size due to the left atrium’s asymmetrical anatomical shape. In this study, a new index (modified-LA/Ao ratio) is developed to measure the left atrium size that combines two different views of the heart. (Modified-LA/Ao ratio = (LA diameter from right parasternal long-axis four-chamber view + LA diameter from right parasternal short-axis view)/Ao diameter)). This study reviewed a total of 136 dogs including both control dogs and myxomatous mitral valve disease dogs and found that our new index showed comparable performance to the traditional method and may be particularly helpful in detecting mild left atrium enlargement in early disease stages. This may help veterinarians detect changes earlier and make better treatment decisions. Our new index is simple to use and may be a helpful tool for monitoring heart disease in dogs.

## 1. Introduction

Currently, echocardiography is the standard method for evaluating left atrial (LA) size in dogs. According to the BENEFIT study, most veterinary echocardiographers preferred linear two-dimensional (2-D) measurements, primarily using the right parasternal short-axis view (RPSV) and indexing LA size to the aortic diameter (LA/Ao ratio). Although the right parasternal long-axis four-chamber view (RPL4CV) was commonly used for subjective assessment, it was infrequently applied for linear measurements [1]. The maximum left atrial dimension (LAD), measured from RPL4CV, has been studied in normal hearts and in cases of myxomatous mitral valve disease (MMVD) [2,3,4,5,6]. In these studies, LAD showed better intraobserver and interobserver variability than the LA/Ao ratio. Veterinarians also commonly use the LAD measured in RPL4CV to assess the LA size in cats and horses [3,7,8,9]. Based on the author’s experience, discrepancies in LA size can occur between different echocardiographic views. Since the LA has an asymmetrical three-dimensional structure, relying on 2-D measurement from only one view can decrease the accuracy in detecting LA enlargement [10,11,12]. In the LA size assessment through radiography, the index measuring the LA in two dimensions has been shown to be more effective in detecting LA enlargement than reflecting only single dimension of LA [13]. The LA/Ao ratio alone or LAD alone demonstrated less accuracy in detecting LA enlargement compared to using both indices together [4]. We propose a new echocardiographic index, the modified (M)-LA/Ao ratio, which utilizes two different views.

This study has two main objectives: (a) to evaluate the ability of the M-LA/Ao ratio to predict severity of mitral valve regurgitation by analyzing its correlation with conventional indices measured from thoracic radiography or echocardiography (vertebral heart score (VHS), vertebral left atrial size (VLAS), modified-VLAS (M-VLAS), LA/Ao ratio, left ventricular internal dimension in diastole normalized for body weight (LVIDdN) and peak velocity of early diastolic transmitral flow (E)), and (b) to assess its ability to differentiate between the MMVD stages according to the American College of Veterinary Internal Medicine (ACVIM) consensus guidelines, using receiver operating characteristic (ROC) curve analysis. This study was based on the hypothesis that the M-LA/Ao ratio, incorporating the two-dimensional assessment of the LA, would provide improved accuracy over conventional indices in staging MMVD.

## 2. Materials and Methods

### 2.1. Animals

This observational retrospective study reviewed radiographic and echocardiographic records in healthy dogs (control group) and dogs diagnosed with MMVD that were presented to Jeonbuk National University Veterinary Medical Teaching Hospital between 5 August 2020 and 9 June 2024. Dogs in the control group were client-owned animals that were presented for routine medical check-ups and showed no evidence of systemic or cardiac disease. Dogs diagnosed with MMVD were also client-owned and were presented either for routine health screening, referral due to a heart murmur detected on auscultation, or clinical signs consistent with pulmonary edema. All owners provided informed consent for the use of their dogs’ data in this study. Signalment data (including age, body weight, breed, and sex) and current medications at the time of examination were recorded. All dogs included in this study underwent a physical examination, blood pressure measurement, thoracic radiography, echocardiography, and the SNAP 4DX-plus test (to exclude heartworm infection) on the same day. MMVD was diagnosed via echocardiography and was defined by myxomatous changes in the mitral valve (thickening and prolapse of the mitral valve) along with mitral regurgitation (MR) during systole as observed on color-flow Doppler. Dogs with systemic diseases that could affect the cardiovascular system or those on medications unrelated to MMVD management were excluded from the study. Dogs with MMVD were strictly classified according to the ACVIM consensus guidelines [14]. All Dogs in MMVD stage B2 met all criteria proposed in ACVIM consensus guidelines. (murmur intensity ≥ 3/6, VHS ≥ 10.5, LA/Ao ratio ≥ 1.6, and LVIDdN ≥ 1.7)

### 2.2. Thoracic Radiography

All radiographs were acquired using the same equipment. (HF-525 Plus VET, Ecoray, Seoul, Republic of Korea) Inspiratory right lateral and ventrodorsal views were acquired in thoracic radiography. In patients with cardiogenic pulmonary edema (CPE), a dorsoventral view was acquired, instead of a ventrodorsal view, depending on the patient’s condition. In such cases, the timing of the inspiratory phase may not have been accurately captured in both views. All thoracic radiographic measurements were taken from the right lateral view, following the details outlined in previous studies [13,15]. The lung fields and clinical signs were also assessed in dogs suspected of having CPE.

### 2.3. Echocardiography

**Figure 1:**
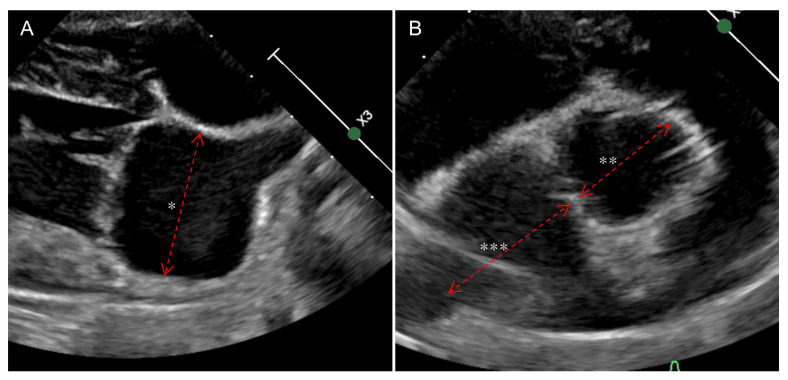
The figures demonstrate the measurements used to calculate the M-LA/Ao ratio. (**A**) illustrates the measurement of LAD (*) in the right parasternal long-axis four-chamber view, where LAD is measured parallel to the mitral valve annulus at the point of maximum LA diameter. (**B**) illustrates the measurements required to calculate the LA/Ao ratio in the right parasternal short-axis view. The aortic diameter (**) is measured along the commissure between the noncoronary cusp and the left cusp. The LA dimension (***) is measured by extending the same line used for the aortic measurement, carefully excluding the pulmonary vein. All measurements are obtained using the inner-edge-to-inner-edge method. LAD—Maximum left atrial dimension, LA/Ao ratio—Left-atrium-to-aorta ratio, M-LA/Ao ratio—Modified-LA/Ao ratio.

All echocardiograms were performed by two trained veterinarians (MS and MW) using the same equipment (EPIC 7C, Koninklijke Philips N.V., Amsterdam, The Netherlands). Echocardiographic images were acquired from both left and right echocardiographic windows, with simultaneous single-lead electrocardiograms. All echocardiographic measurements, including both 2D and M-mode indices, were performed using the endocardial border as the reference, applying the inner-edge-to-inner-edge method. The sedative status of each patient was not recorded. The LA/Ao ratio was measured from the RPSV, at the end of the systole, one frame after the aortic valve closure (Figure 1A) [16]. The LAD was measured from the RPL4CV view using a line parallel to the mitral annulus, measuring the maximum diameter of the left atrium at the end of ventricular systole, one frame before the mitral valve opening. Normalization of LAD to the bodyweight was performed according to the previous study (LADn = LAD(cm)/Body weight(kg)^0.309^) [2]. The left ventricular inner diameter was measured from the RPSV at the end of the diastole, just before the Q wave started, utilizing the M-mode. Normalization of LVIDd was calculated according to the previous study (LVIDdN = LVIDd(cm)/(Body weight(kg))^0.294^) [17]. Peak velocity of early diastolic transmitral flow (E) was measured using the left apical four-chamber view using Doppler echocardiography with pulsed-wave sample volume (2 mm in width) placed between the opening of the mitral valves. The M-LA/Ao ratio was calculated using the following formula: (LAD from RPL4CV + LA diameter from RPSV)/Ao diameter. All echocardiographic views were retrospectively obtained as three second videos, and measurements were taken from three consecutive cycles, with the mean values recorded. Discrepancy between the LA/Ao ratio and LADn was evaluated by applying predefined cutoff values for LA enlargement (LA/Ao ≥ 1.6 and LADn ≥ 1.57). The number of cases with concordant and discordant classifications was summarized descriptively [2,18]. All radiographic and echocardiographic images were reviewed and measured using commercial digital viewing software. (INFINITT DICOM viewer, version 3.0.11, INFINITT Healthcare, Seoul, Republic of Korea)

### 2.4. Inter- and Intraobserver Agreement

For interobserver agreement, eight dogs from this study were randomly selected and the LAD and LA/Ao ratio were measured by four veterinarians (two trained veterinarians (MS, MW), one intern, and the corresponding author). For intraobserver agreement, eight dogs from this study were randomly selected and the LAD and LA/Ao ratio were measured by one trained veterinarian (MS) 48 h apart. All individuals performing the measurements were blinded to the information of the dogs. The selected dogs included four from the control or stage B1 group and four from stage B2 or C, to represent a range of disease severity.

### 2.5. Statistical Analysis

All statistical analyses were performed using a commercial program (IBM SPSS statistics, version 29.0.0.0 (171); SPSS Inc., Chicago, IL, USA). The Kolmogorov–Smirnov test was used to assess the normality of continuous data. Based on the data distribution, the results were reported as the median and interquartile range (IQR). Age, body weight, and other measurements derived from thoracic radiography and echocardiography were compared among the control, stage B1, stage B2, and stage C groups using the Kruskal–Wallis test. Pairwise comparisons between the control and stage B1 groups, the stage B1 and B2 groups, and the stage B2 and C groups were conducted using the Mann–Whitney U test, with the Bonferroni correction applied for multiple comparisons. Additionally, the Mann–Whitney U test was used to compare the cardiac non-remodeling group (control group and stage B1 group) with the cardiac remodeling group (stage B2 and C group). The Spearman correlation test was applied to analyze the relationships between continuous measurements. ROC curve analysis was used to assess the indices (LADn, LA/Ao ratio, and M-LA/Ao ratio) to determine their ability to differentiate between groups. Cutoff value for LADn, LA/Ao ratio (only between B2 and C), and M-LA/Ao ratio were determined by using Youden’s index to differentiate between the stage B1 and B2, as well as between the stage B2 and C. Interobserver and intraobserver variabilities for the LA/Ao ratio and LAD were assessed using intraclass correlation coefficient (ICC) estimates and their 95% confidence intervals (CI) based on a single measurement. An ICC value above 0.9 was considered excellent, 0.75 to 0.9 as good, 0.5 to 0.75 as moderate, and below 0.5 as poor [19]. Statistical significance was set at *p* value < 0.05.

## 3. Results

A total of 136 dogs were included in this study, including 24 in the control group, 52 in the stage B1 group, 27 in the stage B2 group, and 33 in the stage C group. A summary of patient characteristics and medications is presented in Table 1. Age was significantly different between the control and stage B1 groups (*p* < 0.001), while body weight differed significantly different between stage B2 and stage C (*p* = 0.012). The control group consisted of six Beagles, six mixed breeds, three Shih Tzus, two Miniature Schnauzers, two Pomeranians, two Toy Poodles, one Shepherd, one Chihuahua, and one Yorkshire Terrier. The stage B1 group consisted of eleven Malteses, nine mixed breeds, six Toy Poodles, six Miniature Schnauzers, four Cocker Spaniels, three Chihuahuas, three Beagles, two Shih Tzus, two Bichon Frizes, one Pomeranian, and one Pekinegese. The stage B2 group consisted of seven Malteses, four mix breeds, four Pomeranians, three Cocker Spaniels, two Miniature Schnauzers, two Shih Tzus, two Chihuahuas, one Yorkshire Terrier, one Spitz, and one Miniature Pinscher. The stage C group consisted of sixteen Malteses, five mixed breeds, three Chihuahuas, three Shih Tzus, two Pomeranians, two Toy Poodles, one Yorkshire Terrier, and one Miniature Pinscher.

All measurements are summarized in Table 2. In the Spearman correlation (ρ) analysis, M-LA/Ao ratio and LADn showed positive correlations between other measurements. The results of the Spearman correlation (ρ) analysis are summarized in Table 3. The Kruskal–Wallis analysis revealed significant differences between the groups for all indices.

**Figure 2:**
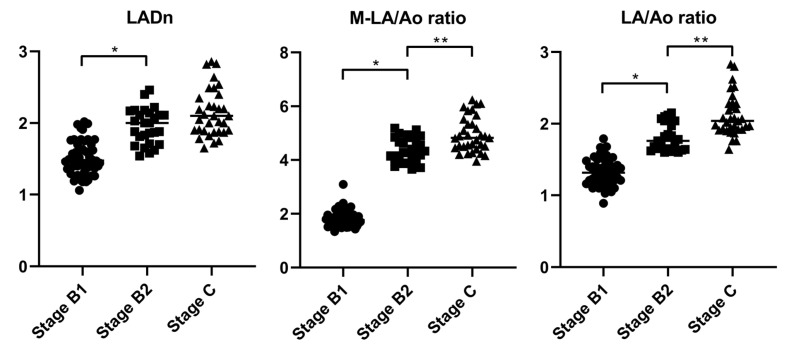
Scatter plots of the LADn, M-LA/Ao ratio and LA/Ao ratio across different ACVIM stages. The bars indicate the median values for each measurement. All measurements were statistically different between stage B1 and stage B2. In the M-LA/Ao ratio and LA/Ao ratio, significant differences were also noted between stage B2 and stage C. ACVIM—American College of Veterinary Internal Medicine, LADn—Normalized maximum left atrial dimension, LA/Ao ratio—Left-atrium-to-aorta ratio, M-LA/Ao ratio—Modified-LA/Ao ratio. An asterisk (*) denotes a statistically significant difference between ACVIM stages B1 and B2, whereas double asterisks (**) denote a statistically significant difference between stages B2 and C.

All measurements were compared sequentially between the groups: control–stage B1, stage B1–stage B2, and stage B2–stage C. VLAS and LADn were significantly different between the control and B1 groups (*p* = 0.01 and *p* = 0.005, respectively). All measurements were significantly different between the stage B1 and stage B2 groups (*p* < 0.001). The LA/Ao ratio and M-LA/Ao ratio were significantly different between stage B2 and C (*p* < 0.001 and *p* = 0.001, respectively). Scatter plots of the LADn, LA/Ao ratio, and M-LA/Ao ratio are shown in Figure 2.

**Figure 3:**
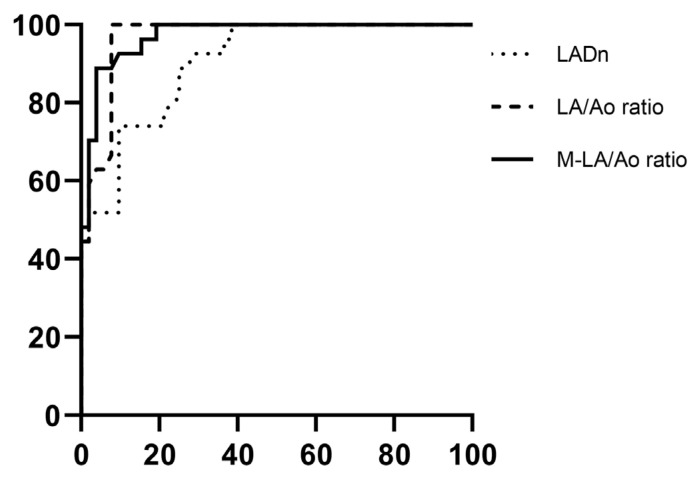
Illustrations of receiver operating characteristic (ROC) curves for LADn, LA/Ao ratio, and M-LA/Ao ratio distinguishing between ACVIM stage B1 and stage B2. The area under the ROC curve (AUC) for LADn was 0.924, for LA/Ao ratio, 0.979, and for M-LA/Ao ratio, 0.981. ACVIM—American College of Veterinary Internal Medicine, LADn—Normalized maximum left atrial dimension, LA/Ao ratio—Left-atrium-to-aorta ratio, M-LA/Ao ratio—Modified-LA/Ao ratio.

ROC curve analysis was performed to differentiate between the stage B1 and stage B2 groups, as well as between the stage B2 and stage C groups, using the LA/Ao ratio, LADn, and M-LA/Ao ratio. (Figure 3) For the stage B1 and stage B2 comparison, the area under the curve (AUC) values were 0.968 (95% confidence interval (CI): 0.934–1.002) for the LA/Ao ratio, 0.904 (95% CI: 0.841–0.968) for LADn, and 0.973 (95% CI: 0.943–1.002) for the M-LA/Ao ratio. In the comparison between stage B2 and stage C, the AUC values were 0.818 (95% CI: 0.700–0.922) for the LA/Ao ratio, 0.664 (95% CI: 0.527–0.801) for LADn, and 0.743 (95% CI: 0.619–0.867) for the M-LA/Ao ratio. The cutoff values distinguishing stage B1 and stage B2 were 3.83 for the M-LA/Ao ratio (sensitivity: 87%, specificity: 96%) and 1.53 for LADn (sensitivity: 100%, specificity: 92%). The cutoff values separating stage B2 from stage C were 1.86 for the LA/Ao ratio (sensitivity: 93%, specificity: 26%) and 4.43 for the M-LA/Ao ratio (sensitivity: 79%, specificity: 39%). The intraobserver and interobserver variabilities, as assessed by ICC, are summarized in Table 4.

Discrepant classification between LA/Ao ratio and LADn was identified in 21 dogs across all stages. Specifically, 2 dogs in the control group showed LADn values above the enlargement threshold despite having normal LA/Ao ratio. Among the 18 dogs in stage B1 with discrepancies, 17 exceeded the LADn cutoff (≥1.57) only, while 1 exceeded the LA/Ao cutoff (≥1.6) only. In the stage B2 group, one dog had a LA/Ao ratio above the cutoff with a normal LADn value.

## 4. Discussion

Because the LA has an asymmetrical shape, assessing its size from a single echocardiographic view may lead to inaccuracies. To address this limitation and incorporate the assessment of the LA from multiple standard views, the authors developed a new echocardiographic index, the modified-LA/Ao (M-LA/Ao) ratio, calculated as (LAD from RPL4CV + LA dimension from RPSV) divided by the aortic diameter from RPSV. The goal of this study was to evaluate the M-LA/Ao ratio for differentiating between ACVIM stages of MMVD and to compare it with other conventional indices.

In the correlation analysis, all conventional indices obtained from thoracic radiography and echocardiography showed positive correlations with M-LA/Ao ratio (*p* < 0.001). The echocardiographic indices showed stronger correlations with each other than with radiographic indices. Among them, parameters assessing LA size demonstrated the highest correlations. These findings suggest that the M-LA/Ao ratio, similar to conventional LA size indices, may serve as a valuable echocardiographic parameter for evaluating LA enlargement in dogs with MMVD. The M-LA/Ao ratio is an echocardiographic index that incorporates two dimensions of the LA, so a positive correlation between the LADn and the M-LA/Ao ratio was an expected outcome.

In the Mann–Whitney analysis, all indices were significantly different between the cardiac non-remodeling group (control and stage B1) and the cardiac remodeling group (stage B2 and stage C), as expected (*p* < 0.001). Between the control and stage B1 groups, VLAS and LADn were significantly different (*p* = 0.01, *p* = 0.005, respectively). Additionally, among the indices analyzed, those related to the LA showed lower *p*-values, while those related to the ventricles tended to have higher *p*-values (VHS: *p* = 0.11, M-VLAS: *p* = 0.04, LA/Ao ratio: *p* = 0.03, LVIDdN: *p* = 0.87, E: *p* = 0.66, M-LA/Ao ratio: *p* = 0.02). This may suggest that LA enlargement is one of the earliest detectable structural changes in the course of MMVD, although this interpretation should be made cautiously, as it is based on cross-sectional comparison and a limited sample size. This aligns with a previous study, which found that in ACVIM stage B1, LA enlargement may be present, but LV enlargement is uncommon [20]. In a study by G. Grosso et al., dogs in ACVIM stage B1 with LA enlargement had a lower median survival time compared to ACVIM stage B1 with no LA enlargement, with a median survival time closer to that of dogs in ACVIM stage B2 [20]. This suggests that, although the ACVIM consensus guidelines recommend medical intervention starting at stage B2, early medical intervention may be considered in dogs at stage B1 with LA enlargement [14,21]. It may be necessary to establish a more granular distinction between stages B1 and B2, in which the M-LA/Ao ratio may be utilized. Between the stage B1 and stage B2 groups, all indices were significantly different, as expected (*p* < 0.001). Between stage B2 and C, the LA/Ao ratio and M-LA/Ao ratio were significantly different. Among the other measurements, only those related to the LA tended to have lower *p*-values (VHS: *p* = 0.42, VLAS: *p* = 0.08, M-VLAS: *p* = 0.02, LADn: *p* = 0.03, LA/Ao ratio: *p* = 0.001, LVIDdN: *p* = 0.56, E: *p* = 0.73, M-LA/Ao ratio: *p* = 0.001). These results suggest that left atrial size may be more reflective of MMVD severity than left ventricular size, in both early and advanced stages of the disease. However, this interpretation should be made with caution, as it is based on cross-sectional data, and statistical significance alone may not fully capture the clinical relevance of each variable. Moreover, the LA/Ao ratio and M-LA/Ao ratio may assist in differentiating CPE from noncardiac causes of pulmonary disease in dogs with cardiac remodeling. There was no significant difference in E between stage B2 and C. A previous study found that E was effective in differentiating congestive heart failure (CHF); however, the study compared CHF versus non-CHF within MMVD patients [22]. Although E is used to assess the severity and prognosis of MMVD patients, the results of this study, along with those of previous studies, suggest that distinguishing CHF within the cardiac remodeling group using E may be challenging [21,23,24].

ROC analysis was performed to compare stage B1 and stage B2, as well as between stage B2 and stage C, using the LADn, LA/Ao ratio, and M-LA/Ao ratio. Between stage B1 and stage B2, all indices assessed in the ROC analysis demonstrated excellent AUC values [25] (LADn: AUC = 0.904, LA/Ao ratio: AUC = 0.968, M-LA/Ao ratio: AUC = 0.973). The cutoff values for distinguishing between stage B1 and stage B2, determined using Youden’s index, were 1.53 for LADn (sensitivity: 100%, specificity: 92%) and 3.83 for M-LA/Ao ratio (sensitivity: 87%, specificity: 96%). Given that the current staging criteria already incorporate the LA/Ao ratio for diagnosing stage B2, a direct comparison between the two indices may introduce bias and should be interpreted with caution. In this study, both the M-LA/Ao ratio and the LA/Ao ratio show excellent and comparable diagnostic performance. However, as shown in the scatter plots in Figure 2, the overlap between stages B1 and B2 is more pronounced with the LA/Ao ratio than with the M-LA/Ao ratio. The M-LA/Ao ratio integrates two different dimensions of LA, providing a more comprehensive estimate of LA size and yielding a wider numerical range than single-view indices. This broader range may offer additional clinical value in detecting mild LA enlargement, particularly in cases where traditional indices show borderline results. In the comparison between stage B2 and stage C, the AUC for LADn was below 0.7, indicating poor diagnostic accuracy; therefore, no cutoff value was determined. This finding differs from the results of F. Marchesotti et al. [3]. This result may be attributed to differences in the number of patients and the scaling exponents used. The cut-off values for the LA/Ao ratio and M-LA/Ao ratio, derived using Youden’s index, were 1.86 (sensitivity: 93%, specificity: 26%) and 4.43 (sensitivity: 79%, specificity: 39%), respectively, with AUC values of 0.83 and 0.76 [25]. Since the comparison between stage B2 and stage C focused on dogs with advanced cardiac remodeling, the weaker correlation between absolute LA size and occurrence of CPE in these cases may explain why both indices demonstrated only limited AUC [12]. The distinction between stages B2 and C is primarily based on the presence of CPE, which may not be directly correlated with absolute LA size. Further comparison between the echocardiographic indices evaluated in this study and more accurate methods for assessing LA volume, such as cardiac MRI, is warranted.

In the assessment of the inter- and intraobserver measurement agreement, the interclass ICC was 0.75 for the LA/Ao ratio and 0.94 for LAD, indicating higher reliability for LAD. Similarly, the intraclass ICC values were 0.93 and 0.99, respectively, with LAD again showing greater reliability. These results are consistent with previous studies [2,3,4,5]. While the LA/Ao ratio is derived from the standard parasternal short-axis view and is widely used in clinical practice, the addition of LAD, measured from a different view, may introduce a slight increase in variability when forming the M-LA/Ao ratio. However, since LAD has demonstrated excellent intra- and interobserver reliability in this and previous studies, we believe the overall reproducibility of the M-LA/Ao ratio remains acceptable for clinical use. Moreover, because the M-LA/Ao ratio incorporates measurements from two different planes, it may better reflect the asymmetric size of the LA, thereby providing a more accurate assessment of LA enlargement [3,6,10,12].

To assess whether relying on a single echocardiographic view introduces discrepancies in evaluating LA enlargement, we applied definitions based on previous studies, using either an LA/Ao ratio > 1.6 or an LADn > 1.56 as cutoff values [2,18]. A discrepancy was considered present if one measurement indicated enlargement while the other remained within the normal range. Discrepancies were observed in 2 dogs from the control group, 18 dogs from the stage B1 group, 1 dog from the stage B2 group, and none in the stage C group. In the majority of discrepant cases, only LADn exceeded the cutoff for LA enlargement, suggesting that LADn may capture subtle changes in LA size that are not reflected by the LA/Ao ratio. Moreover, this suggests that discrepancies between the LADn and the LA/Ao ratio are more likely to occur when LA enlargement is mild, highlighting the asymmetric nature of the LA and the importance of evaluating from multiple views. Additionally, in the stage B1 group, all dogs with discrepancies had an M-LA/Ao ratio lower than the cutoff value for differentiating between stage B1 and stage B2, as suggested in this study. Based on these findings, using the M-LA/Ao ratio to assess LA size may be particularly useful in dogs with MMVD and mild LA enlargement, when discrepancies exist between the LADn and LA/Ao ratio.

This study has several limitations. First, Maltese dogs were overrepresented in the stage B2 and stage C groups, accounting for 7 out of 27 in stage B2 and 16 out of 33 dogs in stage C. The impact of this overrepresentation on the results is unclear, and further studies with larger sample sizes and a more diverse range of breeds or breed-specific research are needed. Second, age was significantly different between the control and stage B1 groups, and body weight was significantly different between the stage B2 and C groups. Variations in age and body weight can influence cardiac measurements and remodeling, although the extent of this effect is unclear. This study was retrospective in nature. The types and dosages of medications administered varied, and whether sedation was used during echocardiography and radiography was not consistently recorded. In cases where sedation was used, butorphanol was the primary agent used, which has minimal effects on echocardiographic measurements [26,27]. Moreover, in cases of acute CPE, it was not documented whether imaging was performed before or after furosemide administration. As furosemide can influence LA pressure, this may have affected the echocardiographic findings [28]. While this study analyzed echocardiographic indices reflecting LA enlargement, such as the LA/Ao ratio and M-LA/Ao ratio, across MMVD stages, it did not confirm whether these indices accurately reflect LA size. Comparing these indices with LA volume measurements obtained using advanced imaging techniques may be necessary to assess their accuracy [11,23,29].

## 5. Conclusions

The M-LA/Ao ratio is an index that reflects two dimensions of the LA and is effective in evaluating LA size in dogs with MMVD, particularly in cases of mild LA enlargement. The M-LA/Ao ratio was developed to evaluate LA size by integrating measurements from two different echocardiographic views. The M-LA/Ao ratio was significantly higher in stage B2 and C groups compared to the control and stage B1 groups. ROC curve analysis identified an optimal cutoff value of ≥3.83 for the M-LA/Ao ratio, with a sensitivity of 87% and a specificity of 96% in distinguishing stage B2 from the combined stage B1 and B2 groups. The M-LA/Ao ratio, which incorporates two-dimensional measurements of the LA, appears to be an effective index for assessing LA size in dogs with MMVD, particularly for detecting early LA enlargement.

## Figures and Tables

**Table 1:** Summarized signalments and medications administered at the time of assessment of patients.

	Control Group	ACVIM Stage B1	ACVIM Stage B2	ACVIM Stage C
Total (*N* = 136)	24	52	27	33
Age (y)	7.0 (4.25–10.5)	11.0 (9.0–13.0) *	11.0 (10.0–12.0)	12.0 (10.0–14.75)
Weight (kg)	6.06 (4.04–10.8)	4.83 (4.01–7.5)	5.19 (3.33–7.69)	3.55 (2.28–5.11) ^§^
Sex (M:F)	9:15	23:29	13:14	19:14
Medications at the time of evaluation	n/a	n/a	Pimobendan (11/27)	Furosemide (15/33) Pimobendan (17/34) Enalapril (10/34) Spironolactone (7/34)

Median (IQR) for continuous data. * Statistically significant difference compared with the control group (*p* < 0.001). ^§^ Statistically significant difference compared to ACVIM stage B2 (*p* = 0.012). ACVIM—American College of Veterinary Internal Medicine, M—Male, F—Female.

**Table 2:** Summary of radiographic and echocardiographic measurements in patients.

	Control	ACVIM Stage B1	ACVIM Stage B2	ACVIM Stage C
VHS	10.3 (10.0–10.7)	10.55 (10.2–11.0)	11.5 (11.0–12.2) ^§^	11.2 (10.6–12.1)
VLAS	2.0 (1.9–2.2)	2.15 (2.0–2.3) *	2.7 (2.5–3.0) ^§^	2.8 (2.5–3.0)
M-VLAS	2.8 (2.7–3.0)	3.1 (2.73–3.4)	3.8 (3.53–4.3) ^§^	4.1 (3.6–5.0)
E (m/s)	0.68 (0.61–0.84)	0.70 (0.59–0.79)	1.15 (0.97–1.35) ^§^	1.20 (0.94–1.57)
LVIDdN	1.46 (1.30–1.57)	1.44 (1.3–1.57)	1.83 (1.7–1.94) ^§^	1.82 (1.73–2.05)
LA/Ao ratio	1.25 (1.10–1.35)	1.32 (1.21–1.43)	1.77 (1.65–2.02) ^§^	2.01 (1.92–2.27) ^¶^
LADn	1.35 (1.24–1.45)	1.48 (1.36–1.68) *	1.88 (1.7–2.14) ^§^	2.07 (1.88–2.32)
M-LA/Ao ratio	1.78 (1.32–2.09)	3.16 (2.91–3.56)	4.3 (3.92–4.84) ^§^	4.84 (4.46–5.46) ^¶^

Median (IQR) for continuous data. * Statistically significant difference compared to the control (VLAS, *p* = 0.01; LADn, *p* = 0.005). ^§^ Statistically significant difference compared to ACVIM stage B1 (*p* < 0.001). ^¶^ Statistically significant difference compared to ACVIM stage B2 (LA/Ao ratio, *p* < 0.001; M-LA/Ao ratio, *p* = 0.001). ACVIM—American College of Veterinary Internal Medicine, VHS—Vertebral heart score, VLAS—Vertebral left atrial size, M-VLAS—Modified-VLAS, E—Peak velocity of early diastolic transmitral flow, LVIDdN—Normalized left ventricular internal dimension in diastole, LA/Ao ratio—Left-atrium-to-aorta ratio, LADn—Normalized maximum left atrial dimension, M-LA/Ao ratio—Modified-LA/Ao ratio.

**Table 3:** Summary of Spearman correlation (ρ) analysis results.

	VHS	VLAS	M-VLAS	LA/Ao Ratio	LVIDdN	E	LADn
M-LA/Ao ratio	0.489	0.663	0.71	0.961	0.799	0.746	0.869

All *p* values for the variables are below 0.001. VHS—Vertebral heart score, VLAS—Vertebral left atrial size, M-VLAS—Modified-VLAS, E—Peak velocity of early diastolic transmitral flow, LVIDdN—Normalized left ventricular internal dimension in diastole, LA/Ao ratio—Left atrium to aorta ratio, LADn—Normalized maximum left atrial dimension, M-LA/Ao ratio—Modified-LA/Ao ratio.

**Table 4:** Intra- and interobserver agreement for maximum left atrial dimension (LAD) and left-atrium-to-aorta ratio (LA/Ao ratio).

	Intraobserver Agreement			Interobserver Agreement		
Measurements	ICC	95% CI	*p* value	ICC	95% CI	*p* value
LAD	0.99	0.97–0.99	<0.001	0.94	0.85–0.98	<0.001
LA/Ao ratio	0.93	0.71–0.98	<0.001	0.75	0.51–0.92	<0.001

ICC—Intraclass correlation coefficient, CI—Confidence interval.

## Data Availability

The data are contained within the article. The data presented in this study are available in the tables and figures in the article “A Novel Echocardiographic Index (Modified-Left-atrium-to-aorta ratio) for Quantifying Left Atrial Size and Differentiating Stages in Dogs with Myxomatous Mitral Valve Disease”.

## Abbreviations

The following abbreviations are used in this manuscript:

ACVIM	American College of Veterinary Internal Medicine
AUC	Area under the curve
CHF	Congestive heart failure
CPE	Cardiac pulmonary edema
ICC	Intraclass correlation coefficient
LA/Ao ratio	Left-atrium-to-aorta ratio
LA	Left atrium
LAD	Maximum left atrial dimension
LADn	Maximum left atrial dimension normalized for body weight
LVIDdN	Left ventricular internal dimension in diastole normalized for body weight
M-LA/Ao ratio	Modified-left-atrium-to-aorta ratio
M-VLAS	Modified-vertebral left atrial size
MMVD	Myxomatous mitral valve disease
ROC	Receiver operating characteristic
RPL4CV	Right parasternal long-axis four-chamber view
RPSV	Right parasternal short-axis view
VHS	Vertebral heart score
VLAS	Vertebral left atrial size
